# Crystal structures of a purple acid phosphatase, representing different steps of this enzyme's catalytic cycle

**DOI:** 10.1186/1472-6807-8-6

**Published:** 2008-01-31

**Authors:** Gerhard Schenk, Tristan W Elliott, Eleanor Leung, Lyle E Carrington, Nataša Mitić, Lawrence R Gahan, Luke W Guddat

**Affiliations:** 1 School of Molecular and Microbial Sciences, The University of Queensland, St. Lucia, QLD 4072, Australia

## Abstract

**Background:**

Purple acid phosphatases belong to the family of binuclear metallohydrolases and are involved in a multitude of biological functions, ranging from bacterial killing and bone metabolism in animals to phosphate uptake in plants. Due to its role in bone resorption purple acid phosphatase has evolved into a promising target for the development of anti-osteoporotic chemotherapeutics. The design of specific and potent inhibitors for this enzyme is aided by detailed knowledge of its reaction mechanism. However, despite considerable effort in the last 10 years various aspects of the basic molecular mechanism of action are still not fully understood.

**Results:**

Red kidney bean purple acid phosphatase is a heterovalent enzyme with an Fe(III)Zn(II) center in the active site. Two new structures with bound sulfate (2.4 Å) and fluoride (2.2 Å) provide insight into the pre-catalytic phase of its reaction cycle and phosphorolysis. The sulfate-bound structure illustrates the significance of an extensive hydrogen bonding network in the second coordination sphere in initial substrate binding and orientation prior to hydrolysis. Importantly, both metal ions are five-coordinate in this structure, with only one nucleophilic μ-hydroxide present in the metal-bridging position. The fluoride-bound structure provides visual support for an activation mechanism for this μ-hydroxide whereby substrate binding induces a shift of this bridging ligand towards the divalent metal ion, thus increasing its nucleophilicity.

**Conclusion:**

In combination with kinetic, crystallographic and spectroscopic data these structures of red kidney bean purple acid phosphatase facilitate the proposal of a comprehensive eight-step model for the catalytic mechanism of purple acid phosphatases in general.

## Background

At least one-third of enzymes characterized require metal ions to function. Roles include electron transfer reactions, oxidations and a plethora of hydrolytic processes [1]. The majority of these enzymes require one or two metal ions for functionality but more complex multinuclear metal clusters also occur. Amongst metalloenzymes binuclear hydrolases form a diverse family with biological functions including signal transduction and cell cycle progression, nucleotide homeostasis and bone metabolism [2-9]. Members of this group of enzymes have evolved into targets for the development of chemotherapeutic agents.

Binuclear metallohydrolases employ variants of the same basic mechanism to catalyze esterolysis of a large number of substrates, in some cases under inversion of stereochemistry, and in others without [2,8,10-13]. In the latter (e.g. alkaline phosphatase [13]) a covalently modified enzyme intermediate is formed upon nucleophilic attack by a reactive amino acid residue. In the former a metal ion-bound water ligand is the proposed nucleophile, but its precise identity has been subject to debate and may vary in different enzymes [2-9,12,14-18]. The majority of binuclear metallohydrolases require the presence of two metal ions for reactivity, although their precise roles in catalysis and/or substrate or product binding have also remained conjectural [2-9,12,14-18]. Note also that some members of this group of enzymes can operate with a single metal ion in the active site (*e.g*. the amino peptidase from *Aeromonas proteolytica *[19], the methionyl aminopetidase from *Escherichia coli *[19] or the metallo-β-lactamase from *Bacillus cereus *[20]. The requirement for particular metal ions and the coordination environments of the metal ions may vary significantly amongst members of this group of enzymes with di-M(II) centers (where M = Zn, Mn, Ni, Co) being most prevalent. Heterovalent centers of the Fe(III)-M(II) form have also been observed in a group of enzymes termed purple acid phosphatases (PAPs) [2,8,9].

PAPs are active in the pH range between 3.0 and 8.0 and have been purified and characterized from a number of mammals and plants [21-28], and PAP-like genes have been identified in a limited number of microorganisms [29]. The animal enzymes are 35 kDa monomers with redox-active Fe(III)-Fe(II/III) centers where only the heterovalent form is catalytically active [30,31]. Proposed biological roles include iron transport, the generation of reactive oxygen species and bone resorption [32]. The latter has made the enzyme a target for the development of anti-osteoporotic drugs [32,33]. Plant PAPs are 110 kDa homodimers, containing Fe(III)-Zn(II) or Fe(III)-Mn(II) centers [21-23,34,35], and a recombinant isoform from sweet potato has been shown to contain a di-iron center [36]. Proposed biological roles for plant PAPs include phosphate metabolism and the generation of reactive oxygen species [37]. The characteristic purple color is due to a charge transfer transition between a tyrosine side-chain and the Fe(III) [38,39].

**Figure 1:**
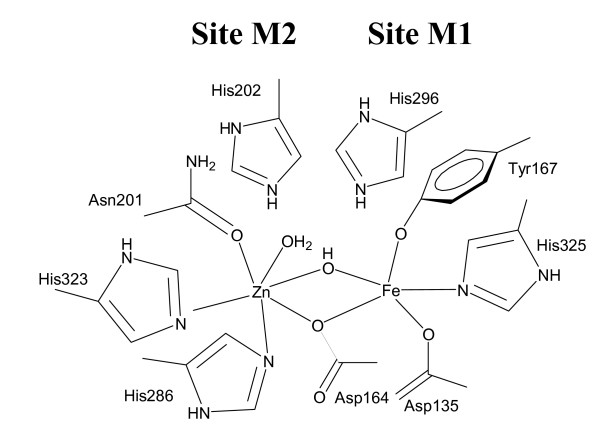
Schematic illustration of the active site of red kidney bean purple acid phosphatase (rkbPAP), a representative binuclear metallohydrolase. In most (if not all) binuclear metallohydrolases the binding affinities of the two metal centers vary, with M1 representing the tight binding site and M2 the lower affinity site [8]. In rkbPAP M1 and M2 are occupied by Fe(III) and Zn(II), respectively. Combined crystallographic and spectroscopic data for PAPs indicate the presence of a bridging (hydr)oxo group and one terminal water ligand (see text). The presence of a terminal Fe(III)-bound hydroxide is currently debated with spectroscopic data suggesting its absence [8], but the crystal structure of rat PAP supporting its presence [8].

The crystal structure of the free red kidney bean PAP (rkbPAP) and complexes with phosphate (both a reaction product and substrate analogue) and tungstate (an inhibitor) are available [40,41]. In addition, structures of phosphate-bound sweet potato PAP [18] and several mammalian PAPs (human, pig, rat) have been determined [42-45]. Despite low overall sequence homology between PAPs from animal and plant sources their active sites are remarkably conserved, with seven invariant metal ligands (Figure 1) [8,9,18,40-47].

In this study the structures of the rkbPAP-sulfate and rkbPAP-fluoride complexes were determined, providing insight into what we propose are two crucial steps in its catalytic mechanism, (i) the pre-catalytic stage, and (ii) the chemical step. In combination with previously reported structural, kinetic and spectroscopic data a comprehensive model for the reaction mechanism involving eight steps is proposed.

## Results

### The structure of the rkbPAP-sulfate complex: a model for a pre-catalytic complex

**Figure 2:**
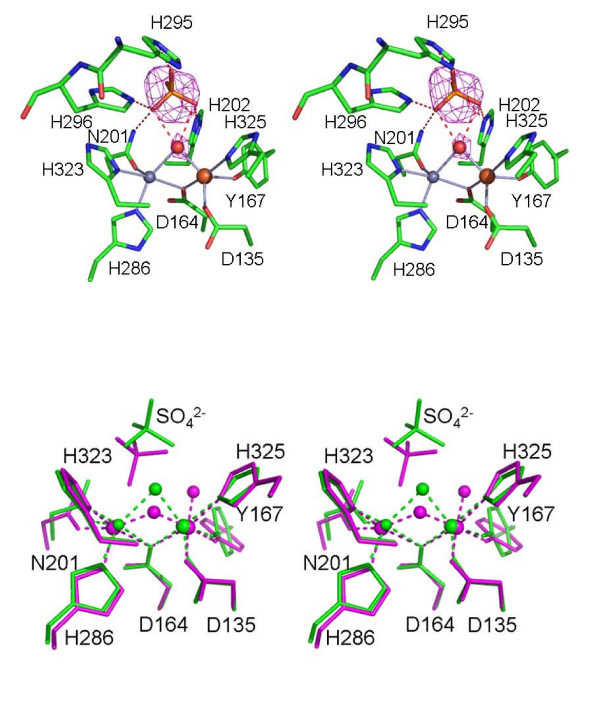
(a) Stereodiagram of the active site of the rkbPAP-sulfate complex. F_o_-F_c _electron density for the sulfate group is overlayed. The sulfate group is bound in the second coordination sphere via extensive hydrogen bonding interactions. General legend: Fe(III) is in tan, Zn(II) in grey, carbon in green, oxygen in red, nitrogen in blue and sulphur in orange. Hydrogen bonds and other contacts are shown as dashed lines. (b) Stereodiagram of the superimposition of the active site of the rkbPAP-sulfate complex (green) with the active site of the rat PAP-sulfate complex [8] (magenta). In the rkbPAP-sulfate complex the bridging hydroxide adopts an elevated position, precluding the binding of a terminal water to Fe(III). Both metals in the rkbPAP-sulfate complex structure are five coordinate.

**Figure 3:**
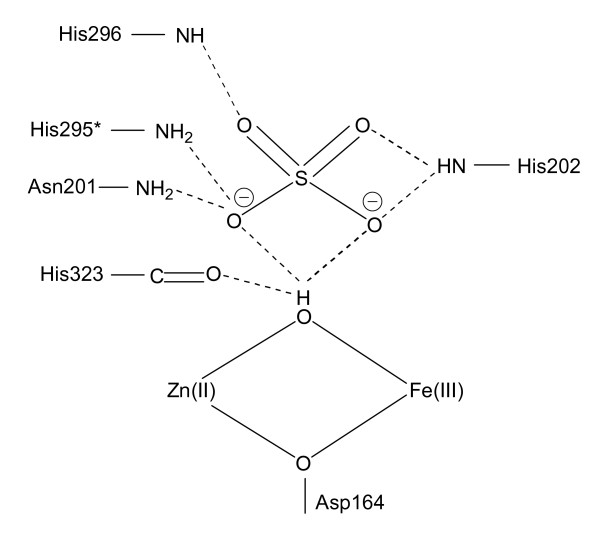
Hydrogen bond network in the active site of rkbPAP. The hydrogen bond pattern demonstrates how the initial, pre-catalytic enzyme-substrate complex may be stabilized. The protonation state of sulfate at the pH of crystallization (pH 4.0) is SO_4_^2-^. Residue His295 (indicated by a "*") is the only amino acid that is not invariant among PAPs from different sources.

In this study rkbPAP was co-crystallized with sulfate, and diffraction data were collected at a resolution (2.4 Å) which is better than that for any previously determined plant PAP structure [18,40,41]. The Fe(III) and Zn(II) in this structure are separated by an average distance of 3.1 Å (Table 1), similar to that reported for the other rkbPAP structures [40,41]. Spherical electron density is observed 2.1 Å from Fe(III) and 2.3 Å from Zn(II) and therefore in a bridging position (Figure 2a). This group is also within 3.1 Å of the carbonyl oxygen of His323, indicating the formation of a hydrogen bond (Figure 3). We therefore assign this density to that of a μ-hydroxo group, an interpretation in agreement with a previous study involving measurements of the magnetization of rkbPAP under catalytically relevant conditions [48]. The Fe(III) is coordinated by a nitrogen atom from the side chain of His325, oxygen atoms from the side chains of the chromophoric Tyr167, Asp135, Asp164 (which bridges both metal ions), and the bridging hydroxide (Figure 2a). These five ligands coordinate the Fe(III) in a trigonal bipyramidal geometry, where Asp135, the bridging hydroxide and Tyr167 are coplanar with Fe(III), and Asp164 and His325 are in axial positions. Zn(II) is also pentacoordinate, ligated by the side-chain oxygen atoms of Asp164 and Asn201, the side-chain nitrogen atoms of His286 and His323 and the μ-hydroxide. Its geometry can be described as distorted square pyramidal (Figure 2a). Mechanistically, it is important to stress that the μ-hydroxide group is the only water molecule present in the active site, and given the location of this group the fitting of a sixth ligand to either metal is implausible (Figure 2b).

**Table 1:** Some interatomic distances (Å).

Atoms		rkbPAP-sulfate	rkbPAP-fluoride
Fe(III)	Zn(II)	3.1	3.5
Fe(III)	OH^-^	2.1	-
Zn(II)	OH^-^	2.3	-
Fe(III)	F^-^	-	2.5
Zn(II)	F^-^	-	2.1
OH^-^	SO_4_^2- ^- O1	2.7	-
OH^-^	SO_4_^2- ^- O3	2.5	-
F^-^	Na^+^	-	2.6
Na^+^	SO_4_^2- ^- O1	-	2.4
Na^+^	SO_4_^2- ^- O3	-	2.6
His323 C = O	OH	3.1	-
His323 C = O	F^-^	-	3.6
Asn201 Nδ2	SO_4_^2- ^- O3	3.2	-
His202 Nε2	SO_4_^2- ^- O1	3.0	2.9
His202 Nε2	SO_4_^2- ^- O2	3.1	-
His295 Nε2	SO_4_^2- ^- O4	3.3	-
His296 Nε2	SO_4_^2- ^- O3	2.9	-

### The structure of the rkbPAP-fluoride complex: insight into the chemical step of the hydrolytic reaction

**Figure 4:**
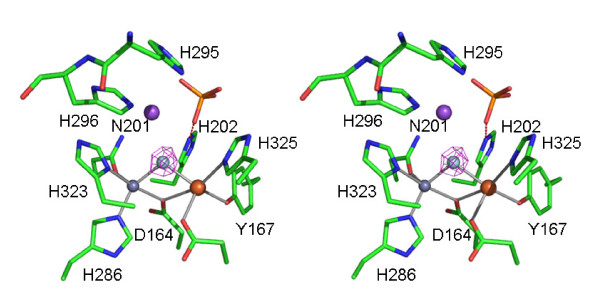
Stereodiagram of the active site of the rkbPAP-fluoride complex. F_o_-F_c _electron density for the bridge is overlayed. The fluoride replaces the hydroxide in the bridging position. General legend: Fe(III) is in tan, Zn(II) in grey, sodium in purple, fluoride in cyan, carbon in green, oxygen in red, nitrogen in blue and sulphur in orange. Hydrogen bonds and other contacts are shown as dashed lines.

rkbPAP incubated with fluoride crystallized in a different space group and with different unit cell parameters compared to the sulfate complex (Table 2). Two dimers in the asymmetric unit are observed yielding four copies of the active site. In these structures the metal ions are on average 3.5 Å apart, a distance significantly larger than that observed in any of the other rkbPAP structures [40,41]. A small sphere of electron density is observed between the two metal ions (Figure 4), in a position similar, but not identical, to that of the μ-hydroxo in the rkbPAP-sulfate complex. The center of the bridging electron density in the rkbPAP-F structure is 2.5 Å and 2.1 Å from Fe(III) and Zn(II), respectively (Table 1). In contrast, the bridging electron density in rkbPAP-SO_4 _is, respectively, 2.1 Å and 2.3 Å from Fe(III) and Zn(II). Thus, the bridging ligand is shifted 0.2 Å closer to the Zn(II) and 0.4 Å further away from the Fe(III); its distance to the carbonyl oxygen of His323 is 3.6 Å, compared to 3.1 Å observed in the rkbPAP-SO_4 _structure (Figure 2). These differences indicate that fluoride rather than μ-hydroxide acts as the bridging ligand, as anticipated from kinetic and spectroscopic data collected for PAPs from pig, human and red kidney bean [15,49,50], and other binuclear metallohydrolases [51]. Additional electron densities in the second coordination sphere, close to the fluoride, are tentatively assigned to a sodium ion and a sulfate ion (see Methods section). The sodium ion may offset the charge of the metal bridging fluoride group, and possibly prevents cations (i.e. sulfate, acetate) from binding to the second coordination sphere.

**Table 2:** Data collection and refinement statistics.

	rkbPAP-sulfate	rkbPAP-fluoride
*Crystal Parameters*		
Unit cell lengths (Å)	*a *= *b *= 148.03 *c *= 160.09	*a *= 85.72 *b *= 188.45 *c *= 192.40
Unit cell angle (°)	*α *= *β *= *γ *= 90.0	*α *= *β *= *γ *= 90.0
Space group	*I*4_1_	*P*2_1_2_1_2_1_
Crystal dimensions (mm)	0.5 × 0.2 × 0.2	0.5 × 0.3 × 0.3
*Diffraction Data*^a^		
Temperature (K)	293	100
Resolution Range (Å)	50.0 – 2.40	50.0 – 2.20
Observations (*I *> 0σ (*I*))	152,476	425,475
Unique reflections (*I *> 0σ (*I*))	55,092	142,147
Completeness (%)	82.0 (52.0)	89.4 (65.1)
*R*_sym_^b^	0.115 (0.302)	0.075 (0.34)
*Refinement*		
^c^*R*_factor_	0.1712	0.2237
*R*_free_	0.2113	0.2543
RMSD^d ^bond lengths (Å)	0.007	0.007
RMSD^d ^bond angles (°)	1.285	1.233
*Ramachandran Plot (%)*		
Most favoured	83.2	83.3
Additionally allowed	15.1	15.1
Generously allowed	1.2	1.2
Disallowed	0.5	0.7

^a^Values in parentheses are for the outer resolution shells. ^b^*R*_sym _= Σ_h _Σ_*i*_|*I*_h,*i *_- <*I*_h_>|/Σ_h _Σ_*i *_*I*_h,*i *_where *I*_h,i _is the intensity of the *i*th measurement of reflection h and <*I*_h_> is the average value over multiple measurements. ^c^*R*_factor _= Σ||F_obs_|-|F_calc_||/Σ|F_obs_|, where the *R*-factor is calculated based on the reflections used in the refinement (90% of the total data) and *R*-free is calculated using the remaining 10% of the data. ^d^RMSD = root mean square deviation.

## Discussion

The study of the reaction mechanism of PAP-catalyzed hydrolysis has received considerable attention over the past decade, with much of the focus directed towards the investigation of individual steps of the catalytic cycle. Various stages of the mechanism have been subject to debate, none more so than the identity of the reaction-initiating nucleophile and the mode of substrate binding [2,3,8,12,14-18,28,35,49,50,52-55]. In this study, we provide insight into these two points of contention by combining the obtained structural data with previously reported spectroscopic, kinetic and crystallographic information, and a comprehensive eight-step model for the reaction cycle is proposed.

### The pre-catalytic phase of the reaction cycle

The rate of the oxidation of reduced pig PAP by [Fe(III)(CN)_6_]^3- ^displays saturation behavior as the concentration of the oxidant is increased [56]. The model proposed to explain the observed saturation behavior invokes an initial rapid formation of an enzyme-[Fe(III)(CN)_6_]^3- ^complex, followed by a considerably slower catalytic step. This conclusion was further augmented by measurements of the rate of phosphate binding to the Fe(III) of pig PAP using stopped-flow spectroscopy, leading to the suggestion that the substrate is also likely to associate initially with the enzyme, forming a catalytically non-competent complex (pre-catalytic complex) [16,56]. It was speculated that interactions between conserved histidine residues in the active site may be responsible for the formation of this pre-catalytic complex (His92 and His195 in pig PAP, corresponding to His202 and His296 in rkbPAP; Figure 1), but the precise mode of binding for this complex remained obscure [16]. X-ray crystallography is an ideal method to visualize bonding interactions between a protein and a ligand. However, in order to study enzyme-substrate interactions suitable substrate analogues need to be available. A possible candidate mimic is phosphate. The crystal structure of rkbPAP with bound phosphate was solved showing that the tetraoxo anion is coordinating to both metal ions (μ-1,3 bidentate mode) [41]. However, since hydrolysis requires the coordination of the substrate to the metal center, followed by a nucleophilic attack by a metal-bound hydroxide [2-12,14-18,28,35,39], the phosphate-bound rkbPAP structure is likely to illustrate the product-bound state rather than providing a mimic for the pre-catalytic substrate-bound complex. The pH for crystallization was 4.5 [41], hence phosphate is likely in its monoanionic form. However, substrates such as *para*-nitrophenolphosphate (*p*NPP) may interact with the enzyme as a dianion [5]. Thus, on the basis of similar charges, sulfate is a more appropriate mimic for the substrate (pK_a2_~2). In contrast to the phosphate-bound rkbPAP structure [41], the sulfate ion in the active site does not coordinate directly to the metal ions. Instead it is positioned in the second coordination sphere, stabilized via an extensive hydrogen bond network involving His202, His295, His296 and the Zn(II) ligand Asn201 (Figures 2 and 3). Hydrogen bonds can also be formed between the μ-hydroxide and two of the sulfate oxygens (note that due to its proximity to three donor oxygens the μ-hydroxide is able to form hydrogen bond interactions with each of these atoms (Figure 3); the crystal structure thus displays the averaged hydrogen bond pattern, evident as a "trifurcated" interaction). Alternatively, it is possible that due to the presence of the sulfate group the pK_a _of the bridging hydroxide is sufficiently elevated so that at pH 4.0 (crystallization condition) it is protonated; in this case each of the protons may point towards one sulfate oxygen atom. At present the two possibilities cannot be distinguished. The intricacies of the extended hydrogen bond network are further illustrated by bi- and trifurcated interactions involving three of the four oxygen atoms of sulfate and several conserved first and second coordination sphere residues of rkbPAP (Figure 3). At pH 4.0 (crystallization condition) sulfate is expected to enter the active site in its dianionic (SO_4_^2-^) form. However, due to its extensive hydrogen bonding interactions the effective charge of sulfate is lowered, reducing its electrostatic attraction for the positively charged metal cluster. Thus, the rkbPAP-sulfate complex demonstrates, for the first time, how the pre-catalytic complex predicted from stopped-flow measurements [16,52,56] may be formed and stabilized in the second coordination sphere of the enzyme. Note, however, that in addition to mimicking the pre-catalytic stage the bound sulfate may also mimic the penultimate phase of catalysis, where the reaction product phosphate is still bound to the active site (see below).

**Figure 5:**
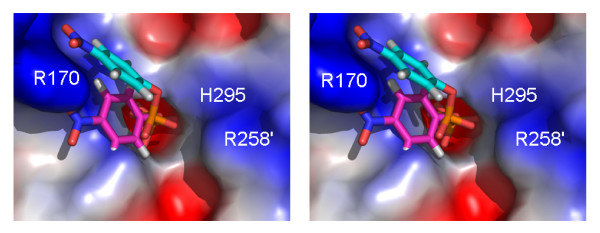
Model of substrate (*para*-nitrophenyl phosphate) binding to rkbPAP based on the sulfate complex (this study; cyan carbons) and on the structure of the phosphate complex (magenta carbons). The ' symbol represents a residue from the neighboring subunit.

In the absence of crystallographic data for the complex between an actual substrate and rkbPAP, *in silico *docking has been used to estimate the most likely binding mode of the leaving group based on the conformation of the sulfate group in the proposed pre-catalytic state (Figure 5). Similarly, the binding mode of the leaving group in the rkbPAP-phosphate complex (which is likely to represent the product-bound state after hydrolysis, see above) was also modeled (see Methods section for details), illustrating the required movement of the substrate in the active site during catalysis.

A sulfate-bound structure has also been reported for rat PAP [43], with a first coordination sphere geometry similar to that of rkbPAP (Figure 2b). However, in contrast to the latter in the rat enzyme SO_4_^2- ^is bound in a monodentate manner to the metal ion at the M2 site (Figure 1) and is stabilized by hydrogen bonding interactions with Asn112 (Asn201) and His113 (His202; residues numbered in brackets indicate the corresponding residues in rkbPAP). A similar mode of sulfate coordination has been observed in the Ser/Thr protein phosphatase from bacteriophage λ (λPP) [57]. Since kinetic and spectroscopic experiments with mammalian PAPs indicate that a monodentate coordination of the substrate to site M2 precedes hydrolysis [12,14,16,17], the rat PAP and λPP structures are thus likely to represent the step following the formation of the pre-catalytic complex (but preceding hydrolysis). The different binding modes of sulfate in rat and rkbPAP may be ascribed to: (1) His216 (His296) in the rat enzyme being prevented from forming a hydrogen bond with sulfate due to the presence of a Zn(II) ion (added in the crystallization buffer); (2) His295 in rkbPAP (Figure 2a) being replaced by Glu215 in rat PAP, thus altering the chemistry at this site; (3) the μ-hydroxide in the rat PAP structure being deprotonated under the conditions of crystallization (pH 7.5); and (4) the di-iron rat enzyme was likely to be crystallized in its inactive diferric form. Thus, in the rat PAP structure the combination of reduced hydrogen bonding interactions and increased positive charge in the metal cluster is likely to increase the electrostatic attraction between the metal center and sulfate, leading to direct metal ion coordination.

### The chemical step of the catalytic cycle

Apart from providing insight into a possible mode of interaction between substrate and enzyme, the rkbPAP-sulfate structure also identifies the metal ion-bridging hydroxide as a candidate nucleophile (Figure 2a). A similar conclusion was reached using a range of spectroscopic techniques, including electron-nuclear double resonance (ENDOR) [17] and X-ray absorption spectroscopy [15].

Fluoride has been shown to inhibit a number of metalloenzymes, and in PAPs is able to replace nucleophilic hydroxides coordinated to the metal ions [15,49-51,58,59]. Specifically, for rkbPAP fluoride is a competitive inhibitor with a K_i _~170 μM at pH 4.90 [50]. The above observation that the bridging hydroxide is the only nucleophile found in the active site of the rkbPAP-SO_4 _crystal structure (Figure 2a) leads to the suggestion that fluoride is able to displace this group, thus forming a μ-fluoro binuclear center. This interpretation is supported by the crystal structure of the rkbPAP-fluoride complex (Figure 4). Importantly, this structure lends credence to an activation mechanism for the hydrolysis-initiating nucleophile. Previous studies proposed a terminal M1-bound hydroxide as the most likely candidate as the nucleophile, and it was suggested that the nucleophilicity of a bridging hydroxide would be too low to assure efficient reactivity [12,14,52]. However, the rkbPAP-SO_4 _structure (see above) and ENDOR measurements of pig PAP [17] indicate that there is no terminal water ligand at the M1 site in the resting and pre-catalytic state (Figures 1 and 3). The latter study [17] suggested that the reduced nucleophilicity of the bridging hydroxide is compensated by an increased electrophilicity of the substrate when comparing the possibilities of (i) a terminally coordinated nucleophile and terminally coordinated substrate, with (ii) a bridging nucleophile and bridging substrate. Furthermore, binding of the substrate decreases the coupling interaction between the two metal centers, indicating a lengthening of the metal-metal distance and a concomitant additional increase of the nucleophilicity of the bridge by effectively shifting it towards the divalent cation ("quasi-monodentate" ligation) [17]. This substrate-induced shift of the bridging ligand is also illustrated for fluoride-bound pig PAP by a combination of inhibition kinetics, resonance Raman, electron paramagnetic resonance and extended X-ray absorption fine structure spectroscopies, which have demonstrated that in the ternary enzyme-fluoride-substrate complex the fluoride ion is located closer to the divalent metal ion [15]. Furthermore, studies involving absorption spectroscopy and (magnetic) circular dichroism have also indicated that the binding of substrate analogues to pig PAP decreases the exchange coupling with an accompanying red-shift of the charge transfer transition [39]. These observations were interpreted in terms of a weakening of the Fe(III)-μ-OH bond. Thus, both μ-fluoride and μ-hydroxide in PAP are affected in a similar manner when substrate binds to the enzyme. Here, the comparison between the sulfate- and fluoride-bound rkbPAP structures corroborates this mechanism for the activation of the bridging nucleophile. The metal-metal distance in rkbPAP-F is elongated by ~0.4 Å and the metal ion-bridging ligand is shifted towards M2 and away from M1 (Table 1). In summary, the combined structural, spectroscopic and kinetic data support the proposal that substrate binding triggers the shift of the μ-hydroxide into a "quasi-terminal" position, thus increasing its nucleophilicity.

### A comprehensive model for the catalytic mechanism

**Figure 6:**
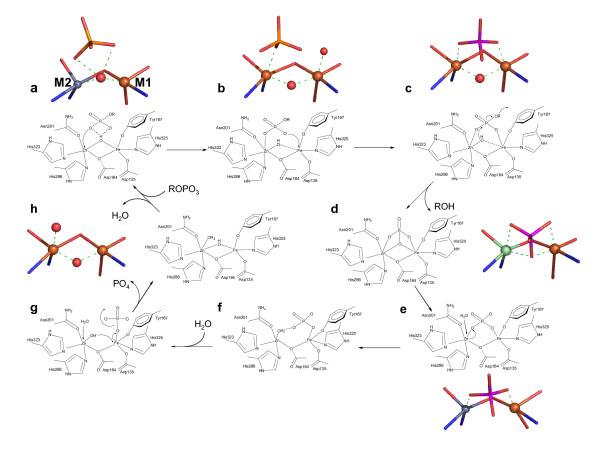
Proposed mechanism of binuclear metallohydrolase-catalyzed esterolysis. Following the binding of the substrate in a pre-catalytic complex, structural rearrangements lead to "quasi-monodentate" and bidentate coordination of the μ-hydroxide and phosphate groups, respectively (*a*-*c*). Nucleophilic attack by the μ-hydroxide is followed by the release of the leaving group, and the active site is returned to its resting state by the exchange of the bound phosphate group by two water molecules (*d*-*h*). A terminal M1-bound hydroxide is observed in the structure of rat PAP (*b*), which appears to be an artefact of crystallization [46] and is not supported by solution studies on resting PAP [17]. Where available crystallographic pictures of relevant active site structures are included. (*a*) rkbPAP-sulfate complex; (*b*) rat PAP-sulfate complex [43]; (*c*) pig PAP-phosphate complex [42]; (*d*) sweet potato PAP-phosphate complex [18] ; (*e*) rkbPAP-phosphate complex [41]; (*h*) rkbPAP [40] (the bridging and terminal M2-bound water ligands were modelled based on ENDOR studies [17]).

Based on all of the available data for PAPs the following model of a comprehensive mechanism of catalysis emerges (Figure 6). Monitoring the oxidation (and thus inactivation) of Fe(III)-Fe(II) pig PAP by [Fe(CN)_6_]^3- ^has provided evidence for the formation of a pre-catalytic complex in the initial phase of the reaction, where the phosphate group of the substrate does not directly coordinate to the metal ions [16,52,56]. The structure of rkbPAP-SO_4 _demonstrates a plausible model for the pre-catalytic complex where the substrate mimic, sulfate, is bound in the second coordination sphere, an arrangement stabilized via an extensive hydrogen bonding network (Figure 3). The bridging μ-hydroxide appears to play an essential role in the initial binding and orientation of the substrate (Figure 2a). In this pre-catalytic state both metal ion sites are five-coordinate with distorted trigonal-bipyramidal geometry. The only solvent molecule in the active site is the μ-hydroxide. Since an ENDOR study provided evidence for the presence of an additional water ligand bound terminally to the metal ion in the M2 site in resting pig PAP (Figure 1) [17], the formation of the pre-catalytic complex may thus lead to the expulsion of this labile terminal water ligand (Figure 6a).

The formation of the pre-catalytic complex with the concomitant expulsion of the M2 site bound terminal water ligand generates a vacant coordination position for an oxygen atom of the phosphate group (monodentate coordination), as exemplified in sulfate-bound structures of rat PAP and λPP (Figure 6b) [43,57]. Coordination of the substrate to the M2 site and subsequent structural rearrangements in the M1 site permit the formation of a μ-1,3 substrate complex (Figure 6c). In support of this hypothesis EPR measurements have indicated that, in fluoride-inhibited pig PAP, substrates and the reaction product phosphate bind in a similar metal ion-bridging μ-1,3 mode to the binuclear center [15]. The resulting μ-1,3 phosphate complex, visualized in the structure of oxidized (inactive) pig PAP [42], places the phosphorus atom in an ideal position for a nucleophilic attack by the μ-hydroxide moiety (Figure 6c).

The proposed structural rearrangements in the M1 site are likely to be mediated via the hydrogen bond network in the second coordination sphere. A related observation has been made in the mononuclear soybean lipoxygenase-1 where the degree of coordination flexibility mediated via a hydrogen bond network in the second coordination sphere directly correlates with the reactivity of this enzyme [60]. Superposition of the rkbPAP structures with bound sulfate, fluoride and phosphate demonstrates that the geometry of the protein ligands in the M2 site and the position of the M2 metal ion (Zn) are, within experimental error, fixed. However, for the M1 site in the fluoride bound structure the metal ion is shifted by up to 0.4 Å when compared to rkbPAP-SO_4 _and rkbPAP-PO_4 _which then results in compensatory rotations of the side chains of Tyr167 and His325.

**Figure 7:**
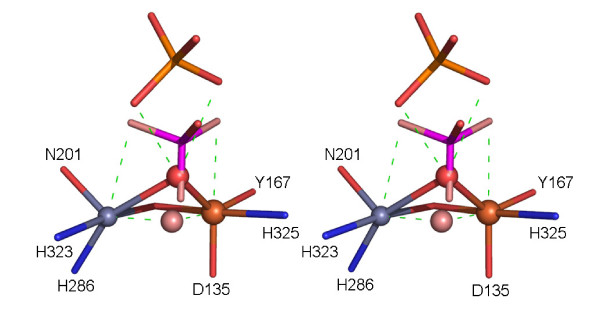
Overlay of the rkbPAP-sulfate complex (with the bridging oxygen in red), the bridging hydroxide (pink) as observed in pig PAP [42] and the phosphate as observed in sweet potato PAP [18]. The image demonstrates a plausible trajectory for the substrate with the bridging hydroxide as the nucleophile in catalysis. Inversion of configuration around the phosphorous atom is observed. General legend: Fe(III) is in tan, Zn(II) in grey, carbon in green, oxygen in red, nitrogen in blue, sulphur in orange, hydrogen in white and phosphorous in magenta.

Nucleophilic attack by the μ-hydroxide and esterolysis of the substrate (depending on the basicity of the leaving group these steps may occur in a concerted or sequential manner [18]) leaves the phosphate bound to the active site in a tripodal geometry (Figure 6d). This mode of coordination has been observed in the crystal structures of sweet potato PAP and di-Ni(II) urease, both with bound phosphate, and di-Mn(II) λPP, with bound sulfate [18,57,61]. Hydrogen bond interactions with the carbonyl oxygen of the metal ion ligand His323 may stabilize this tripodal arrangement, at least at low pH [18]. In Figure 7 the movement required for the phosphate group of the substrate from initial binding in the pre-catalytic complex to the tripodal arrangement following hydrolysis is illustrated.

At this point it is important to point out that evidence has recently emerged that suggests that the chemical step may be affected by both the substrates used in the reaction and the metal ion composition of the active site [53-55]. Specifically, it could be shown that both pig and rkbPAP hydrolyze both ester bonds in the diester substrate methyl-*p*NPP in a processive manner [53]. This observation is interpreted in terms of an initial monodentate coordination of the substrate to the M2-site, followed by a nucleophilic attack by a terminal Fe(III)-bound hydroxide. This process is also observed in reactions catalyzed by a Fe(III)Zn(II) biomimetic of PAPs, where only the terminal hydroxide is sufficiently nucleophilic to induce hydrolysis [62]. Subsequently, but only in the enzyme-catalyzed reaction, the bridging hydroxide initiates the cleavage of the second ester bond of the substrate in a manner similar to that described in the preceding paragraph [53]. Since no terminal Fe(III)-bound hydroxide appears to be present in the resting state of PAPs (see above) the interaction between the diester substrate and the enzyme active site may lead to the introduction of an additional water ligand.

The regeneration of the resting form of the enzyme, which requires the removal of the phosphate from the active site, is currently not well understood. A plausible sequence involves the rearrangement of the bound phosphate group from tripodal (Figure 6d) to μ-1,3 coordination (Figure 6e) via a rotation around the axis formed by the two oxygen atoms of phosphate that are terminally coordinated to the metal ions in sites M1 and M2. Although we are not aware of a precedent where the simultaneous loss of two Lewis interactions is invoked as part of a catalytic mechanism, the resulting μ-1,3-PO_4 _complex has been observed in the structure of rkbPAP, where a μ-hydroxide bridge was also modelled into the active site although this ligand could not be identified in the 2.7 Å electron density map [41]. It is speculated that the loss of strain is a major contributing force to drive the conversion from a tripodal to a μ-1,3 coordination mode. No experimental data for the subsequent steps in regeneration are yet available but a possible mechanism is depicted in Figures 6f–6h. The exchange of the M2-coordinated phosphate oxygen atom by water leads to a monodentate, M1-bound phosphate. Due to geometric constraints the M2-bound water ligand is likely to form a hydrogen bond with the phosphate group (Figure 6f), thus facilitating its deprotonation. The M2-hydroxide then interacts with the metal ion in the M1 site, thus regenerating the μ-hydroxide bridge, weakening the M1-phosphate bond, and providing a vacant coordination position for another water molecule in the M2 site (Figure 6g). The subsequent release of the phosphate group enables the M1 site to regain resting state, trigonal-bipyramidal geometry (Figure 6h). Although the precise details of the phosphate release mechanism remain unknown it is conceivable that the anion is shuttled away from the active site via the second coordination sphere, where it may interact through hydrogen bonds with histidine residues in a manner analogous to that of the substrate in the pre-catalytic complex (Figure 2a). It appears thus likely that the reversible interactions between the phosphate group and the second coordination sphere trigger conformational changes that take place during a catalytic turnover in the active site of PAPs.

## Conclusion

By a combination of crystallographic, kinetic and spectroscopic data an eight-step model for the reaction mechanism of rkbPAP-catalyzed hydrolysis has been developed. Modifications of this basic mechanism are anticipated depending on the substrates used. The mechanistic model developed here may also be relevant for several other binuclear enzymes from the binuclear metallohydrolase family. The observed binding mode for sulfate provides a new lead in the design of metallohydrolase-selective therapeutics.

## Methods

### Materials

All reagents were analytical grade and from Sigma.

### Protein purification and characterization

rkbPAP was purified as described previously [21] concentrated to 25 mg/mL and stored at 4°C in 0.5 M NaCl. Samples for crystallization were > 95% pure, as judged by SDS-PAGE analysis. Metal analysis was carried out using inductively coupled plasma mass spectrometry and indicated the presence of 1.0 Fe, 0.9 Zn, and trace amounts of Mn and Cu per active site.

### Protein crystallization

Crystals were grown by vapor diffusion, with hanging drops consisting of 5 μl of well solution and 5 μl of the concentrated protein solution. For the sulfate complex, the well solution for crystallization consisted of 2 M ammonium sulfate and 0.1 M sodium acetate, pH 4.0. Crystals of the fluoride complex were obtained by co-crystallization of rkbPAP in the presence of 50 mM NaF (an excess of ~550 fold). The well solution for these crystals consisted of 2.3 M ammonium sulfate and 0.1 M sodium acetate pH 4.0. No suitable condition for cryoprotection could be found for the sulfate crystals, therefore data were obtained at room temperature. The fluoride crystal was cryoprotected in a solution containing 30% (v/v) glycerol and 70% (v/v) well solution.

### Diffraction data collection and structural refinement

All X-ray data were collected using an Raxis IV^++ ^imaging plate and an FR-E rotating anode generator operated at a current of 45 mA and a voltage of 45 kV. The program Crystalclear 1.3.6 [63] was used for the integration and scaling of the data. The sulfate and fluoride bound crystals were not isomorphous with any of the previously determined structures of rkbPAP or with each other. Therefore, these structures were solved by molecular replacement in the program EPMR [64] using the polypeptide coordinates of the rkbPAP-phosphate complex (PDB code 4KBP [41]). Rounds of refinement and modelling were undertaken using the programs CNS [65] and O [66], respectively.

The crystal structures of rkbPAP in the presence of sulfate or fluoride were determined by molecular replacement and refined to 2.4 Å with an R_free_value of 0.2113 and to 2.2 Å with an R_free _of 0.2543, respectively (Table 2). The sulfate complex crystallized with one dimer in the asymmetric unit while the fluoride complex has two such dimers. The overall structures of the polypeptides strongly resemble those of the previously determined structures of free rkbPAP and the phosphate and tungstate bound complexes of this enzyme [41] with r.m.s.d. values for all Cα atoms ranging from 0.2 to 0.3 Å. For the sulfate-bound structure the asymmetric unit (and final model) consisted of two polypeptide subunits of 423 residues (9–432), two Fe(III), two Zn(II), two sulfate groups, eight N-acetyl glucosamine residues and 399 water molecules. The first eight N-terminal amino acid residues of the polypeptide in each subunit were not visible. For the fluoride structure the asymmetric unit (and final model) consisted of four polypeptide subunits of 423 residues (9–432), four Fe(III), four Zn(II), four fluoride ions, eight sodium ions, eight sulfate groups, sixteen N-acetyl glucosamine residues and 547 water molecules. Within the crystallization solution, the only other small molecules present at a detectable concentration are acetate and glycerol. The fitting of these molecules into the electron density occupied by the sulfate ions was tested but they did not fit the contours as precisely as sulfate. Furthermore, in F_o_-F_c _difference maps the electron density at the location of the sulfate atoms was generally between 7.5–8.0 σ above the mean, compared to 3.0–4.0 σ above the mean for other nearby atoms which we assign as either oxygen, fluoride or sodium. The backbone dihedral angles (Ramachandran plot values) of His 323, Ile 340 and Ala 243 are in disallowed regions in all polypeptides of both complexes. Only His323 is within the active site. In all of the rkbPAP structures previously determined this residue has similar dihedral angle values [40,41]. Coordinates and structure factors have been deposited into the protein databank with accession numbers 2QFR and 2QFB for rkbPAP-sulfate and rkbPAP-fluoride, respectively. All figures were generated with the progam PyMOL [67].

For *in silico *docking studies a three dimensional model of the substrate *para*-nitrophenyl phosphate was constructed using Sketcher in Insight2000. The phosphorous and the oxygen atoms of the substrate were superimposed onto sulfate in the rkbPAP-sulfate complex or phosphate in the rkbPAP-phosphate [41] complex. In both dockings the aromatic ring of *para*-nitrophenyl phosphate was then rotated to make optimal contacts with the nearby amino acids of the enzyme.

## Abbreviations

ENDOR – electron-nuclear double resonance; λPP – Ser/Thr protein phosphatase from bacteriophage λ; PAP – purple acid phosphatase; rkbPAP – red kidney bean purple acid phosphatase; *p*NPP – *para*-nitrophenolphosphate

## Authors' contributions

All authors have read and approved the final version of the manuscript. GS was responsible for the conceptualization of experiments, analysis and interpretation of data, and drafting and critical review of the manuscript. TWE carried out protein purification, acquisition and analysis of data. EL was assisting acquisition and analysis of data. NM was involved in analysis of data and was responsible for the revision of the manuscript. LEC was involved in protein purification and acquisition of data. LRG was involved in conceptualization of experiments, interpretation of data, and critical review of the manuscript. LWG was responsible for the conceptualization of experiments, acquisition, analysis and interpretation of data, and critical review of the manuscript.

## References

[B1] Lippard SJ, Berg JM (1994).

[B2] Wilcox DE (1996). Binuclear metallohydrolases. Chem Rev.

[B3] Dismukes GC (1996). Manganese enzymes with binuclear active sites. Chem Rev.

[B4] Barford D, Das AK, Egloff MP (1998). The structure and mechanism of protein phosphatases: Insights into catalysis and regulation. Annu Rev Biophys Biomol Struct.

[B5] Jackson MD, Denu JM (2001). Molecular reactions of protein phosphatases – Insights from structure and chemistry. Chem Rev.

[B6] Lowther WT, Matthews BW (2000). Structure and function of the methionine aminopeptidases. Biochimica Et Biophysica Acta-Protein Structure and Molecular Enzymology.

[B7] Solomon EI, Brunold TC, Davis MI, Kemsley JN, Lee SK, Lehnert N, Neese F, Skulan AJ, Yang YS, Zhou J (2000). Geometric and electronic structure/function correlations in non-heme iron enzymes. Chem Rev.

[B8] Mitic N, Smith SJ, Neves A, Guddat LW, Gahan LR, Schenk G (2006). The catalytic mechanisms of binuclear metallohydrolases. Chem Rev.

[B9] Sträter N, Lipscomb WN, Klabunde T, Krebs B (1996). Two-metal ion catalysis in enzymatic acyl- and phosphoryl-transfer reactions. Angewandte Chemie-International Edition in English.

[B10] Mueller EG, Crowder MW, Averill BA, Knowles JR (1993). Purple Acid-Phosphatase – a Diiron Enzyme That Catalyzes a Direct Phospho Group Transfer to Water. J Am Chem Soc.

[B11] Wynne CJ, Hamilton SE, Dionysius DA, Beck JL, Dejersey J (1995). Studies on the Catalytic Mechanism of Pig Purple Acid-Phosphatase. Arch Biochem Biophys.

[B12] Merkx M, Pinkse MWH, Averill BA (1999). Evidence for nonbridged coordination of p-nitrophenyl phosphate to the dinuclear Fe(III)-M(II) center in bovine spleen purple acid phosphatase during enzymatic turnover. Biochemistry.

[B13] O'Brien PJ, Herschlag D (2002). Alkaline phosphatase revisited: Hydrolysis of alkyl phosphates. Biochemistry.

[B14] Aquino MAS, Lim JS, Sykes AG (1994). Mechanism of the Reaction of Different Phosphates with the Iron(Ii)Iron(Iii) Form of Purple Acid-Phosphatase from Porcine Uteri (Uteroferrin). Journal of the Chemical Society-Dalton Transactions.

[B15] Wang XD, Ho RYN, Whiting AK, Que L (1999). Spectroscopic characterization of a ternary phosphatase-substrate-fluoride complex. Mechanistic implications for dinuclear hydrolases. J Am Chem Soc.

[B16] Twitchett MB, Schenk G, Aquino MAS, Yiu DTY, Lau TC, Sykes AG (2002). Reactivity of M-II metal-substituted derivatives of pig purple acid phosphatase (Uteroferrin) with phosphate. Inorg Chem.

[B17] Smoukov SK, Quaroni L, Wang XD, Doan PE, Hoffman BM, Que L (2002). Electron-nuclear double resonance spectroscopic evidence for a hydroxo-bridge nucleophile involved in catalysis by a dinuclear hydrolase. J Am Chem Soc.

[B18] Schenk G, Gahan LR, Carrington LE, Mitić N, Valizadeh M, Hamilton SE, de Jersey J, Guddat LW (2005). Phosphate forms an unusual tripodal complex with the Fe-Mn center of sweet potato purple acid phosphatase. Proc Natl Acad Sci USA.

[B19] Bennett B, Antholine WE, D'Souza VM, Chen GJ, Ustinyuk L, Holz RC (2002). Structurally distinct active sites in the copper(II)-substituted aminopeptidases from Aeromonas proteolytica and Escherichia coli. J Am Chem Soc.

[B20] Wommer S, Rival S, Heinz U, Galleni M, Frere JM, Franceschini N, Amicosante G, Rasmussen B, Bauer R, Adolph HW (2002). Substrate-activated zinc binding of metallo-beta-lactamases – Physiological importance of the mononuclear enzymes. J Biol Chem.

[B21] Beck JL, McConachie LA, Summors AC, Arnold WN, de Jersey J, Zerner B (1986). Properties of a Purple Phosphatase from Red Kidney Bean – a Zinc-Iron Metalloenzyme. Biochim Biophys Acta.

[B22] Schenk G, Ge YB, Carrington LE, Wynne CJ, Searle IR, Carroll BJ, Hamilton S, de Jersey J (1999). Binuclear metal centers in plant purple acid phosphatases: Fe-Mn in sweet potato and Fe-Zn in soybean. Arch Biochem Biophys.

[B23] Durmus A, Eicken C, Sift BH, Kratel A, Kappl R, Huttermann J, Krebs B (1999). The active site of purple acid phosphatase from sweet potatoes (Ipomoea batatas) – Metal content and spectroscopic characterization. Eur J Biochem.

[B24] Campbell HD, Dionysius DA, Keough DT, Wilson BE, de Jersey J, Zerner B (1978). Iron-Containing Acid-Phosphatases – Comparison of Enzymes from Beef Spleen and Pig Allantoic Fluid. Biochem Biophys Res Commun.

[B25] Ketcham CM, Baumbach GA, Bazer FW, Roberts RM (1985). The Type-5, Acid-Phosphatase from Spleen of Humans with Hairy-Cell Leukemia – Purification, Properties, Immunological Characterization, and Comparison with Porcine Uteroferrin. J Biol Chem.

[B26] Averill BA, Davis JC, Burman S, Zirino T, Sandersloehr J, Loehr TM, Sage JT, Debrunner PG (1987). Spectroscopic and Magnetic Studies of the Purple Acid-Phosphatase from Bovine Spleen. J Am Chem Soc.

[B27] Ek-Rylander B, Bill P, Norgard M, Nilsson S, Andersson G (1991). Cloning, Sequence, and Developmental Expression of a Type-5, Tartrate-Resistant, Acid-Phosphatase of Rat Bone. J Biol Chem.

[B28] Funhoff EG, Klaassen CHW, Samyn B, Van Beeumen J, Averill BA (2001). The highly exposed loop region in mammalian purple acid phosphatase controls the catalytic activity. Chem Bio Chem.

[B29] Schenk G, Korsinczky MLJ, Hume DA, Hamilton S, de Jersey J (2000). Purple acid phosphatases from bacteria: similarities to mammalian and plant enzymes. Gene.

[B30] Wang DL, Holz RC, David SS, Que L, Stankovich MT (1991). Electrochemical Properties of the Diiron Core of Uteroferrin and Its Anion Complexes. Biochemistry.

[B31] Bernhardt PV, Schenk G, Wilson GJ (2004). Direct electrochemistry of porcine purple acid phosphatase (uteroferrin). Biochemistry.

[B32] Oddie GW, Schenk G, Angel NZ, Walsh N, Guddat LW, de Jersey J, Cassady AI, Hamilton SE, Hume DA (2000). Structure, function, and regulation of tartrate-resistant acid phosphatase. Bone.

[B33] Valizadeh M, Schenk G, Nash K, Oddie GW, Guddat LW, Hume DA, de Jersey J, Burke TR, Hamilton S (2004). Phosphotyrosyl peptides and analogues as substrates and inhibitors of purple acid phosphatases. Arch Biochem Biophys.

[B34] Beck JL, de Jersey J, Zerner B, Hendrich MP, Debrunner PG (1988). Properties of the Fe(Ii)-Fe(Iii) Derivative of Red Kidney Bean Purple Phosphatase – Evidence for a Binuclear Zn-Fe Center in the Native Enzyme. J Am Chem Soc.

[B35] Schenk G, Boutchard CL, Carrington LE, Noble CJ, Moubaraki B, Murray KS, de Jersey J, Hanson GR, Hamilton S (2001). A purple acid phosphatase from sweet potato contains an antiferromagnetically coupled binuclear Fe-Mn center. J Biol Chem.

[B36] Waratrujiwong T, Krebs B, Spener F, Visoottiviseth P (2006). Recombinant purple acid phosphatase isoform 3 from sweet potato is an enzyme with a diiron metal center. FEBS J.

[B37] Leung E, Teixeira M, Guddat LW, Mitić N, Schenk G (2007). Structure, function and diversity of purple acid phosphatases. Curr Topics Plant Biol.

[B38] Antanaitis BC, Aisen P, Lilienthal HR (1983). Physical Characterization of 2-Iron Uteroferrin – Evidence for a Spin-Coupled Binuclear Iron Cluster. J Biol Chem.

[B39] Yang YS, McCormick JM, Solomon EI (1997). Circular dichroism and magnetic circular dichroism studies of the mixed-valence binuclear non-heme iron active site in uteroferrin and its anion complexes. J Am Chem Soc.

[B40] Sträter N, Klabunde T, Tucker P, Witzel H, Krebs B (1995). Crystal-Structure of a Purple Acid-Phosphatase Containing a Dinuclear Fe(Iii)-Zn(Ii) Active-Site. Science.

[B41] Klabunde T, Strater N, Fröhlich R, Witzel H, Krebs B (1996). Mechanism of Fe(III)-Zn(II) purple acid phosphatase based on crystal structures. J Mol Biol.

[B42] Guddat LW, McAlpine AS, Hume D, Hamilton S, de Jersey J, Martin JL (1999). Crystal structure of mammalian purple acid phosphatase. Structure.

[B43] Lindqvist Y, Johansson E, Kaija H, Vihko P, Schneider G (1999). Three-dimensional structure of a mammalian purple acid phosphatase at 2.2 angstrom resolution with a mu-(hydr)oxo bridged di-iron center. J Mol Biol.

[B44] Uppenberg J, Lindqvist F, Svensson C, Ek-Rylander B, Andersson G (1999). Crystal structure of a mammalian purple acid phosphatase. J Mol Biol.

[B45] Sträter N, Jasper B, Scholte M, Krebs B, Duff AP, Langley DB, Han RL, Averill BA, Freeman HC, Guss JM (2005). Crystal structures of recombinant human purple acid phosphatase with and without an inhibitory conformation of the repression loop. J Mol Biol.

[B46] Schenk G, Guddat LT, Ge Y, Carrington LE, Hume DA, Hamilton S, de Jersey J (2000). Identification of mammalian-like purple acid phosphatases in a wide range of plants. Gene.

[B47] Flanagan JU, Cassady AI, Schenk G, Guddat LW, Hume DA (2006). Identification and molecular modeling of a novel, plant-like, human purple acid phosphatase. Gene.

[B48] Gehring S, Fleischhauer P, Behlendorf M, Huber M, Lorösch J, Haase W, Dietrich M, Witzel H, Locke R, Krebs B (1996). Magnetic susceptibility studies on the diiron forms of the metalloprotein purple acid phosphatase from bovine spleen and kidney bean. Inorg Chim Acta.

[B49] Dikiy A, Funhoff EG, Averill BA, Ciurli S (2002). New insights into the mechanism of purple acid phosphatase through H-1 NMR spectroscopy of the recombinant human enzyme. J Am Chem Soc.

[B50] Elliott TE, Mitić N, Gahan LR, Guddat LW, Schenk G (2006). Inhibition studies of purple acid phosphatases: implications for the catalytic mechanism. J Braz Chem Soc.

[B51] Cama E, Pethe S, Boucher JL, Han SF, Emig FA, Ash DE, Viola RE, Mansuy D, Christianson DW (2004). Inhibitor coordination interactions in the binuclear manganese cluster of arginase. Biochemistry.

[B52] Twitchett MB, Sykes AG (1999). Structure, properties and reactivity of the (FeFeIII)-Fe-II and (ZnFeIII)-Fe-II purple acid phosphatases. Eur J Inorg Chem.

[B53] Cox RS, Schenk G, Mitić N, Gahan LR, Hengge AC (2007). Diesterase Activity and Substrate Binding in Purple Acid Phosphatases. J Am Chem Soc.

[B54] Smith SJ, Casellato A, Hadler KS, Mitić N, Riley MJ, Bortoluzzi AJ, Szpoganicsz B, Schenk G, Neves A, Gahan LR (2007). The reaction mechanism of the Ga(III)Zn(II) derivative of uteroferrin and corresponding biomimetics. J Biol Inorg Chem.

[B55] Schenk G, Peralta RA, Batista SC, Bortoluzzi AJ, Szpoganics B, Dick AK, Herrald P, Hanson GR, Szilagyi RK, Riley MJ, Gahan LR, Neves A (2008). Probing role of the divalent metal ion in uteroferrin using metal ion replacement and a comparison to isostructural biomimetics. J Biol Inorg Chem.

[B56] Aquino MAS, Sykes AG (1994). Redox Reactivity of the Binuclear Iron Active-Site of Porcine Purple Acid-Phosphatase (Uteroferrin). J Chemical Soc Dalton Trans.

[B57] Voegtli WC, White DJ, Reiter NJ, Rusnak F, Rosenzweig AC (2000). Structure of the bacteriophage lambda Ser/Thr protein phosphatase with sulfate ion bound in two coordination modes. Biochemistry.

[B58] Pinkse MWH, Merkx M, Averill BA (1999). Fluoride inhibition of bovine spleen purple acid phosphatase: Characterization of a ternary enzyme-phosphate-fluoride complex as a model for the active enzyme-substrate-hydroxide complex. Biochemistry.

[B59] Funhoff EG, de Jongh TE, Averill BA (2005). Direct observation of multiple protonation states in recombinant human purple acid phosphatase. J Biol Inorg Chem.

[B60] Schenk G, Neidig ML, Zhou J, Holman TR, Solomon EI (2003). Spectroscopic characterization of soybean lipoxygenase-1 mutants: the role of second coordination sphere residues in the regulation of enzyme activity. Biochemistry.

[B61] Benini S, Rypniewski WR, Wilson KS, Ciurli S, Mangani S (2001). Structure-based rationalization of urease inhibition by phosphate: novel insights into the enzyme mechanism. J Biol Inorg Chem.

[B62] Neves A, Lanznaster M, Bortoluzzi AJ, Peralta RA, Casellato A, Castellano EE, Herrald P, Riley MJ, Schenk G (2007). An Unprecedented FeIII(μ-OH)ZnII Complex that Mimics the Structural and Functional Properties of Purple Acid Phosphatases. J Am Chem Soc.

[B63] Pflugrath JW (1999). The finer things in X-ray diffraction data collection.. Acta Crystallogr D Biol Crystallogr.

[B64] Kissinger CR, Gehlhaar DK, Fogel DB (1999). Rapid automated molecular replacement by evolutionary search. Acta Crystallogr D Biol Crystallogr.

[B65] Brünger AT, Adams PD, Clore GM, DeLano WL, Gros P, Grosse-Kunstleve RW, Jiang JS, Kuszewski J, Nilges M, Pannu NS, Read RJ, Rice LM, Simonson T, Warren GL (1998). Crystallography & NMR system: A new software suite for macromolecular structure determination. Acta Crystallogr D Biol Crystallogr.

[B66] Jones TA, Zou JY, Cowan SW, Kjeldgaard M (1991). Improved Methods for Building Protein Models in Electron-Density Maps and the Location of Errors in These Models. Acta Crystallogr A.

[B67] DeLano WL (2002).

